# The Sale of Snuff in Atlanta

**Published:** 1886-07

**Authors:** 


### Editorial.

### THE SALE OF SNUFF IN ATLANTA.

Some weeks ago the *Constitution* contained a statement that Atlanta was the third largest snuff market in the world, there being sold here annually more than three hundred thousand pounds.

The statement is astounding. Three hundred thousand pounds of snuff sold annually in Atlanta! Not for foreign export, but for retail, in the environment of which she is the commercial center.

Who buys it? What becomes of it?

Many articles are used to adulterate more costly commodities. He is a lucky man who gets pure butter on his table; quantities of lard are found in the market of which the American hog is guiltless; large amounts of sweet oil and wine tempt the buyer, which the vine and the olive did not yield; chalk baryta and talc are under various forms made to substitute wholesome and nourishing substances. But it is not possible to employ snuff to adulterate anything. If taken at all it must be taken straight. Few are ever seen using it, and no one will confess the practice, yet 300,000 pounds of it are annually made way with. Smoking, chewing and snuffing—the three ways in which the domestic use of tobacco is practiced—were all doubtless transported to and imitated in Europe from the continent of America. Smoking was popularized by the example of the cultured and elegant Sir Walter Raleigh; chewing, like the sturdy savages where it originated, made its own way among all classes of men without patronage or extraneous encouragement. Catharine de Medicis established and made fashionable the custom of snuff-taking in fashionable society, where it long held an honored place. A costly snuff-box was the most welcome present between kings and gentlemen, and the delicate fingers of ladies were often seen conveying the titillating powder to Grecian noses. This rather elegant custom has fallen into "innocuous disuetude." The beautiful snuff-boxes have disappeared with the refined and charming old ladies who used them, and with the ruffle-shirted old Virginia gentleman, who, with stately steps and courtly bow, asked the honor of testing the odor of their contents.

In a score of years now an observer will scarcely meet a snuff-taker or see a snuff-box. What, then, becomes of the three hundred thousand pounds of snuff sold in Atlanta? Men do not consume it; they smoke, it is true, and chew *ad nauseam*, and consume fabulous quantities of tobacco in these methods, but they are so untutored that they could not gracefully perform the convulsive sternutatory motions excited by the presence of a single pinch of snuff in their noses. As no animal upon the earth will touch it, it follows that the immense domestic consumption of this article is made by women.

When and how is it done? No one ever sees it; it cannot be after the manner of our grandmothers; a single jeweled snuff-box would hold enough to supply her elegant necessities for a dozen years.

It may not be prudent to say more, but the tongue of scandal has not been silent, and incredibly shocking stories are told and damaging suspicions entertained of women; modest Christian women stealthily cramming spoonfuls of the dirty, fœtid, poisonous stuff into their beautiful mouths, and rubbing it there with tongue and brush and finger until the flood of saliva, made turbid by the mixture, flows over lips and chin and cheek and raiment to the unutterable disgust of a chance beholder and of all who credit the story.

